# Schlafen family member 11 indicates favorable prognosis of patients with head and neck cancer following platinum-based chemoradiotherapy

**DOI:** 10.3389/fonc.2022.978875

**Published:** 2023-01-19

**Authors:** Seijiro Hamada, Satoshi Kano, Junko Murai, Takayoshi Suzuki, Nayuta Tsushima, Takatsugu Mizumachi, Masanobu Suzuki, Tsuyoshi Takashima, Daiki Taniyama, Naoya Sakamoto, Yoichiro Fujioka, Yusuke Ohba, Akihiro Homma

**Affiliations:** ^1^ Department of Otolaryngology-Head and Neck Surgery, Faculty of Medicine and Graduate School of Medicine, Hokkaido University, Sapporo, Japan; ^2^ Institute for Advanced Biosciences, Keio University, Tsuruoka, Japan; ^3^ Department of Molecular Pathology, Graduate School of Biomedical and Health Sciences, Hiroshima University, Hiroshima, Japan; ^4^ Department of Cell Physiology, Faculty of Medicine and Graduate School of Medicine, Hokkaido University, Sapporo, Japan

**Keywords:** head and neck squamous cell carcinoma, immunohistochemistry, platinum-based chemoradiotherapy, prognostic marker, Schlafen family member

## Abstract

Recently, Schlafen family member 11 (SLFN11) has been reported to increase the sensitivity of cancer cells to DNA-damaging agents, including platinum derivatives; thus, SLFN11 may be a predictive biomarker for platinum-based chemoradiotherapy (CRT). In this study, we examined whether SLFN11 expression was associated with the therapeutic outcome of platinum-based CRT in head and neck squamous cell carcinoma (HNSCC). We performed immunohistochemical analyses for SLFN11 expression in 161 HNSCC tissues from patients who had been administered cisplatin-based CRT and examined the correlation between SLFN11 expression and progression-free survival (PFS). Additionally, SLFN11 expression was examined in 10 paired samples obtained before and after CRT in patients with local failure. Furthermore, *in vitro* experiments were performed using several HNSCC cell lines and isogenic *SLFN11*-knockout cells to assess the association between SLFN11 expression and drug sensitivity. PFS was found to be significantly better in the SLFN11-positive group than in the SLFN11-negative group among the 161 patients (5-year PFS: 78.8% vs. 52.8%, respectively, *p* < 0.001). Similar results were observed for the PFS at each primary site. The percentage of SLFN11 positivity was lower in tumor samples from patients with local failure after CRT than that in the corresponding primary tumors before CRT in 8 of 10 cases. Results of the *in vitro* assay demonstrated that *SLFN11*-knockout cells exhibited reduced sensitivity to DNA-damaging agents but not to the non-DNA-damaging agent docetaxel. Our findings suggest that SLFN11 may serve as a potential biomarker for predicting the response of HNSCC patients to platinum-based CRT.

## 1 Introduction

The principal curative treatments for head and neck squamous cell carcinoma (HNSCC) include surgery, radiation, chemotherapy, or combination therapy. Surgery is the standard of care for HNSCC but often results in large functional and cosmetic burdens, such as total laryngectomy. Platinum (cisplatin or carboplatin)-based chemoradiotherapy (CRT) is an alternative to surgery for locally advanced cases and remains the mainstay of conservative treatments, even with the availability of new classes of anticancer drugs such as molecular targeted drugs and immune checkpoint inhibitors. Cisplatin is a well-known anticancer agent that exerts cytotoxic effects by crosslinking purine bases on the DNA strand. Cisplatin–DNA adducts activate various signaling pathways, including ataxia telangiectasia mutated and Rad3-related (ATR), p53, p73, and mitogen-activated protein kinase pathways, which induce apoptosis (1). Although cisplatin has been a key drug for treating HNSCC for decades, methods for overcoming acquired resistance or resistance to cisplatin are lacking. Moreover, there are no clinical biomarkers available for predicting the response to cisplatin in any cancer type, including HNSCC.

Recently, Schlafen family member 11 (SLFN11) was discovered as a predictive biomarker for anticancer agents, including platinum derivatives, topoisomerase inhibitors, DNA synthesis inhibitors, and poly (ADP-ribose) polymerase (PARP) inhibitors (2–4). The word “Schlafen” in German means “to sleep”; Schlafen genes were first discovered in mouse lymphoid tissues with G_0_/G_1_ cell cycle arrest, which enables proper thymus development (5). SLFN11 is a member of the human Schlafen family (SLFN5, 11, 12, 13, and 14). SLFN11 has 901 amino acid residues and harbors an RNA-binding domain at its N-terminus and a helicase domain at its C-terminus (6). We previously reported that SLFN11 binds to stressed replication forks through replication protein A, and irreversibly blocks replication, independent of ATR protein kinase-mediated checkpoint regulation (7, 8). Clinical studies on ovarian, colorectal, gastric, and lung cancers revealed that high expression of SLFN11 is positively correlated with high sensitivity to platinum derivatives (3, 9–11). However, the utility of SLFN11 as a biomarker for predicting cisplatin sensitivity in HNSCC is undetermined. In this study, we examined the utility of SLFN11 expression in HNSCC *via* retrospective clinical data analysis, immunohistochemical analysis of biopsy samples, and *in vitro* cell viability assays.

## 2 Materials and methods

### 2.1 Patients

We retrospectively reviewed patients with HNSCC treated with definitive cisplatin-based concurrent CRT at Hokkaido University Hospital, Japan, between 2008 and 2019. Patients treated with chemotherapy other than cisplatin, induction chemotherapy, or curative surgery prior to CRT were excluded from the analysis. All patients were treated with either intra-arterial cisplatin (dose of 100–120 mg/m^2^ per week for 4–7 weeks) or intravenous cisplatin (dose of 40 mg/m^2^ per week for 5–6 weeks). Additionally, all patients received external radiotherapy (4 or 6 MV photons from a linear accelerator) to the primary sites and regional lymphatic area. The total radiation dose was 64–70 Gy (median, 70 Gy), which was administered in 32–35 fractions of 2 Gy over 6 weeks. We proposed CRT with intra-arterial cisplatin infusion for patients with locally advanced cancers of the nasal cavity and paranasal sinuses, the base of the tongue, and the larynx or hypopharynx (unilateral). The clinical stages of all patients were evaluated according to the 7^th^ edition of the American Joint Committee on Cancer/Union for International Cancer Control TNM staging system. We differentiated p16-positive from p16-negative oropharyngeal cancer (OPC), as many studies have shown that patients with p16-positive OPC have a significantly better prognosis than those with p16-negative OPC (12–15). This study was conducted in accordance with the Declaration of Helsinki and Good Clinical Practice guidelines. The study protocol was approved by the Institutional Review Board of Hokkaido University Hospital (No. 020-0055). Written informed consent was obtained from the patients whom we could contact. We applied an opt-out method for patients lost to follow-up and for deceased patients. Information on the study was disclosed on the study website so that patients or their representatives could refuse enrollment.

### 2.2 Immunohistochemistry

Biopsy samples from before CRT were available for all enrolled patients (161 cases in total). Immunohistochemical analysis was performed using formalin-fixed, paraffin-embedded tissue specimens as described previously (16). The primary antibodies used were mouse monoclonal anti-SLFN11 antibody (D-2, #sc-515071, special request, 2 mg/mL, Santa Cruz Biotechnology, Dallas, TX, USA), rabbit monoclonal anti-Ki-67 antibody (SP6, #418071, Nichirei Biosciences, Inc., Tokyo, Japan), and mouse monoclonal anti-p53 antibody (DO-7, #413231, Nichirei Biosciences, Inc.). The anti-SLFN11 antibody was diluted at 1:500 in Tris-buffered saline containing 0.05% polyoxyethylene sorbitan monolaurate before use.

### 2.3 Evaluation of immunohistochemistry results

The staining intensity in the immunohistochemical analysis was evaluated independently by three authors (one pathologist [D.T.] and two head and neck surgeons [S.H. and T.S.]) who were blinded to the clinical data. Positively stained cells in the tumor areas of the tissue sections were counted at high magnification (×200). The median value of SLFN11 positivity was 15%; hence, SLFN11 positive staining was defined as ≥15% staining of the tumor nuclei. The median value of Ki-67 positivity was 40%; hence, we defined a positive result as ≥40% staining of the tumor nuclei. For p53 expression, a three-point scale was applied: complete confluent negative staining was considered to indicate extremely negative expression, strong diffuse confluent positivity was considered to indicate extremely positive expression, and intermediate expression of any intensity was considered to be non-extreme expression (17–19). Aberrant expression of p53, i.e., extremely positive or negative expression, corresponds with the p53 mutation status in ovarian and breast cancers (18, 20). Additionally, aberrant expression of p53 is significantly associated with poor tumor control in breast cancer and salivary duct carcinoma (18, 19). Therefore, we analyzed both extremes to assess p53 status.

### 2.4 Cell lines

The human HNSCC cell lines HSC-2, HSC-3, and HSC-4 were obtained from the Japanese Collection of Research Bioresources cell bank (Osaka, Japan). SCC-9 and SCC-25 cells were obtained from the American Type Culture Collection (Manassas, VA, USA). The prostate cancer cell line DU145 was obtained from the NCI Division of Cancer Treatment and Diagnosis (Bethesda, MD, USA). HSC-2, HSC-3, and HSC-4 cells were cultured in Dulbecco’s modified Eagle medium (DMEM; #D5796, Sigma, St. Louis, MO, USA) supplemented with 10% fetal bovine serum (FBS; #FBS-12A, Capricorn Scientific, Ebsdorfergrund, Germany) at 37°C in a humidified atmosphere of 5% CO_2_. SCC-9 and SCC-25 cells were cultured in DMEM/Nutrient Mixture F12 medium (containing L-glutamine and sodium bicarbonate without HEPES; the medium was liquid, sterile-filtered, and suitable for cell culture; DMEM/F12; #D8062, Sigma) supplemented with 10% FBS and 400 ng/mL hydrocortisone (#088-02483, Fujifilm Wako Pure Chemical Corporation, Osaka, Japan) at 37°C in a humidified atmosphere of 5% CO_2_. DU145 cells were cultured in RPMI medium containing 10% FBS at 37°C in a humidified atmosphere of 5% CO_2_.

### 2.5 Generation of SLFN11-knockout cells

*SLFN11*-KO cells were generated using CRISPR/Cas9 in HSC-3 and HSC-4 cells (HSC-3-KO and HSC-4-KO, respectively). Briefly, pTOPO-SLFN11 targeting vector B and pX330-B were co-transfected into the cells using electroporation as described previously (4). After transfection, the cells were released into drug-free medium for 48 h, followed by puromycin selection until single-cell colonies had formed. These colonies were expanded, and gene KO was confirmed by immunoblotting.

### 2.6 RNA isolation and quantitative polymerase chain reaction

Total RNA was isolated from the cells using RNeasy Mini Kit (QIAGEN, Hilden, Germany) according to the manufacturer’s instructions. First-strand cDNA was synthesized from 3.5 μg of total RNA using SuperScript III reverse transcriptase (Invitrogen, Carlsbad, CA, USA). Quantitative reverse transcription PCR was performed using a Light Cycler 480 and Universal Probe Library system (Roche, Basel, Switzerland). The primer sequences were as follows: *SLFN11*-forward, 5′-AACGCCCGATAACCTTCACA-3′; *SLFN11*-reverse, 5′-CTAAGGGGAGGCCCACTAGA-3′; glyceraldehyde-3-phosphate dehydrogenase (*GAPDH*)-forward, 5′-TGGACTCCACGACGTACTCA-3′; and *GAPDH*-reverse, 5′-AATCCCATCACCATCTTCCA-3′. Data were normalized to the *GAPDH* abundance in control cells.

### 2.7 Immunoblotting

Cells were lysed in a solution containing 10 mM Tris-HCl (pH 7.4), 5 mM ethylenediaminetetraacetic acid (EDTA), 150 mM NaCl, 10% glycerol, 1% Triton X-100, 1% sodium deoxycholate, 0.1% sodium dodecyl sulfate (SDS), 50 mM NaF, 1 mM Na_3_VO_4_, and complete (EDTA-free) protease inhibitor (Roche) for 30 min on ice and clarified by microcentrifugation at 15 000 ×*g* for 10 min at 4°C. The supernatants were separated by SDS-polyacrylamide gel electrophoresis and then transferred to polyvinylidene fluoride membranes (Bio-Rad Laboratories, Hercules, CA, USA). The membranes were incubated with primary antibodies (anti-SLFN11, 1:10 000; anti-β-actin [#sc-47778, Santa Cruz Biotechnology], 1:1000), followed by incubation with horseradish peroxidase-labeled secondary antibody (anti-mouse IgG; Jackson ImmunoResearch Laboratories, West Grove, PA, USA). Signals were developed using enzymatic chemiluminescence western blotting detection reagents (#1059243 and #1059250, GE Healthcare, Little Chalfont, UK) and detected using an MIIS imaging system (Givetechs, Sakura, Japan).

### 2.8 Cell viability assays

Cells were seeded into 96-well plates at a density of 5000 cells/well. After 24 h, the original medium was replaced with complete medium containing different concentrations of the following drugs: cisplatin (#AG-CR1-3590-M050, Funakoshi Frontiers In Life Science, Tokyo, Japan) at 100, 50, 25, 12.5, 6.25, 3.13, 1.56, and 0.78 μM; carboplatin (#033-25231, Fujifilm Wako Pure Chemical Corporation) at 1000, 500, 250, 125, 62.5, 31.3, 15.6, 7.81, 3.91, 1.95, and 0.98 μM; olaparib (#CS-0075, Funakoshi Frontiers In Life Science) at 200, 100, 50, 25, 12.5, 6.25, 3.13, and 1.56 μM; docetaxel (#047-31281, Fujifilm Wako Pure Chemical Corporation) at 10, 5, 2.5, 1.25, 0.63, 0.31, 0.16, 0.08, 0.04, 0.02, and 0.01 nM. After 48 or 72 h, cell viability was determined using the Cell Counting Kit-8 (#CK04, Dojindo Molecular Technologies, Kumamoto, Japan) according to the manufacturer’s protocol. The absorbance at 450 nm was measured using a SpectraMax i3x Multi-Mode microplate reader (Molecular Devices, Sunnyvale, CA, USA). The half-maximal inhibitory concentration (IC_50_) of the drug was calculated by fitting the dose-response curves to a four-parameter, variable slope sigmoid dose-response model using the ImageJ software (version 1.53f, NIH, Bethesda, MD, USA).

### 2.9 Statistical analysis

Two-tailed Fisher’s exact test was used to analyze the association between SLFN11 status and the following clinicopathological factors: age, sex, Karnofsky Performance Status, primary site, clinical stage, primary tumor stage, lymph node involvement, histological tumor differentiation, cisplatin injection methods, and Ki-67 and p53 expression. Progression-free survival (PFS) was defined as the time from treatment initiation to disease progression, such as residual or recurrence disease, or death from any cause. Overall survival (OS) was defined as the time from treatment initiation to death from any cause. PFS and OS curves were drawn using the Kaplan–Meier method and compared using the log-rank test. A univariate Cox proportional hazards model was used to evaluate predictive factors for PFS and OS. Variables with *p* < 0.1 in univariate Cox proportional hazards analysis were entered into a multivariate Cox proportional hazards model. The Wilcoxon signed-rank test was used to compare differences in the percentage of SLFN11 and Ki67 positivity between primary tumors before CRT and tumors showing local failure after CRT. Statistical significance was set at *p* < 0.05. *In vitro* experiments were performed at least in triplicate, and the data are expressed as the mean ± standard error of the mean. Statistical calculations were performed using R software (Version 4.0.3, The R Project for Statistical Computing, Vienna, Austria).

## 3 Results

### 3.1 SLFN11 positivity and clinicopathological characteristics

**Figure 1:**
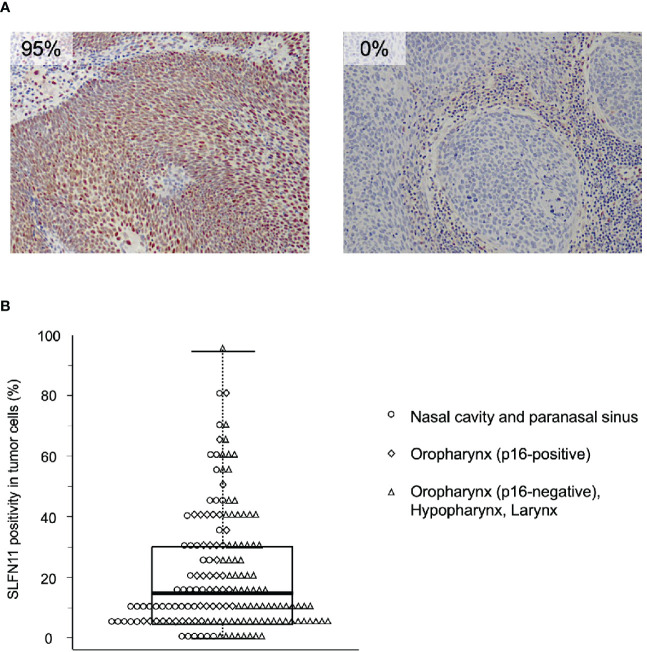
Immunohistochemical examination of Schlafen family member 11 (SLFN11) in head and neck squamous cell carcinoma (HNSCC). **(A)** Representative images of immunohistochemical staining of SLFN11 in HNSCC. Formalin-fixed, paraffin-embedded tissue specimens of tumor biopsy samples before chemoradiotherapy (CRT) were subjected to immunohistochemistry using an anti-SLFN11 antibody. Left, an example of an SLFN11-positive case, in which most tumor cell nuclei were positive for SLFN11 (SLFN11: 95%). Right, an example of an SLFN11-negative case, in which no SLFN11-positive cells were observed in the tumor area (SLFN11: 0%). Stromal cell staining was not counted. **(B)** Dot and box-and-whisker plots showing the distribution of the percentage of SLFN11 positivity. Each dot represents the value for an individual case, whose primary site is indicated on the right. The highest and lowest boundaries of the box represent the 25^th^ and 75^th^ percentiles, respectively, and the whiskers above and below the box designate the maximum and minimum values, respectively. The line within the box indicates the median value.

Based on the inclusion criteria, 161 patients were enrolled in the study. In total, 69 patients were treated with intra-arterial cisplatin and 92 patients were treated with intravenous cisplatin. The median follow-up time of survivors was 5.36 years (range 1.11–10 years). As SLFN11 is exclusively localized in the nucleus (16), SLFN11-positive cells were distinctly recognized by the pattern of nuclear staining (**Figure 1A**). Vascular endothelial cells, macrophages, and a portion of tumor-infiltrating lymphocytes were positive for SLFN11 (16); therefore, we carefully excluded these cells from counting to properly assess SLFN11 positivity in tumor cells. SLFN11 positivity exhibited broad diversity, ranging from 0% to 95% (**Figure 1B**). The median value of SLFN11 positivity was 15%; hence, we defined a positive result as ≥15% of the tumor nuclei stained, regardless of the intensity. Of the 161 patients with HNSCC, 81 (50.3%) were positive for SLFN11 and 80 (49.7%) were negative for SLFN11. There were no significant associations between SLFN11 positivity and any clinicopathological factors (**Table 1**). Among patients with OPC, SLFN11 positivity was significantly higher in p16-positive cases than in p16-negative cases (*p* = 0.030, **Supplementary Table 1**).

**Table 1:** Clinicopathological characteristics of patients according to SLFN11 status.

Variables	No. of patients			
	All	SLFN11-positive	SLFN11-negative	*p* value
Age (Median = 63 years)				
<63	81	45	36	0.209
≥63	80	36	44	
Sex				
Male	134	66	68	0.674
Female	27	15	12	
KPS				
90, 100	145	74	71	0.609
70, 80	16	7	9	
Primary site				
Nasal cavity and paranasal sinus	43	22	21	0.308
Oropharynx (p16-positive)	40	24	16	
Oropharynx (p16-negative), Hypopharynx, Larynx	78	35	43	
Clinical stage				
II	24	12	12	1
III, IV	137	69	68	
T classification				
T1–2	63	32	31	1
T3–4	98	49	49	
N classification				
N0	78	42	36	0.432
N1–3	83	39	44	
Histological grade				
Well	45	19	26	0.429
Moderate	63	33	30	
Poor	53	29	24	
Cisplatin injection				
Intra-arterial	69	33	36	0.634
Intravenous	92	48	44	
Ki-67				
Negative	65	30	35	0.424
Positive	96	51	45	
p53				
NE	57	28	29	0.870
EP, EN	104	53	51	

KPS, Karnofsky Performance Status; NE, non-extreme; EP, extreme positive; EN, extreme negative.

### 3.2 The SLFN11-positive group exhibited better PFS than the SLFN11-negative group

**Figure 2:**
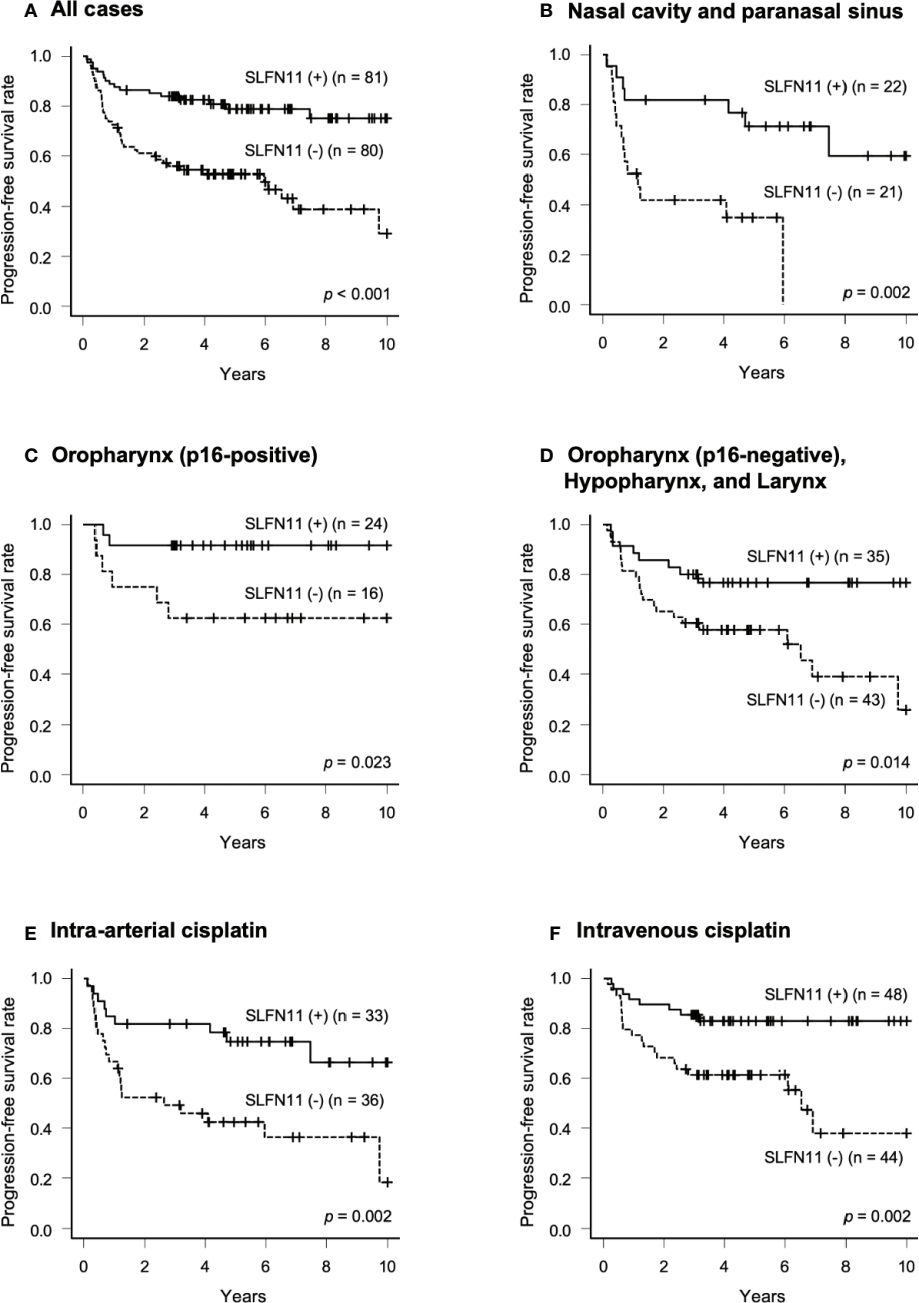
Progression-free survival (PFS) in patients with SLFN11-positive HNSCC. PFS curves generated by the Kaplan–Meier method are shown for all 161 patients in **(A)** and those with tumors arising from each primary site are indicated at the top of the panel in **(B–D)**. Intra-arterial and intravenous cisplatin were analyzed separately in **(E, F)**. The difference between groups was calculated using the log-rank test.

We compared PFS between the SLFN11-positive and SLFN11-negative groups. The PFS of the patients in the SLFN11-positive group was significantly better than that in the SLFN11-negative group (5-year PFS: 78.8% vs. 52.8%, respectively, *p* < 0.001, **Figure 2A**). Next, we performed an in-depth analysis of the primary sites. In nasal cavity and paranasal sinus cancers, p16-positive OPC, and p16-negative oropharyngeal and hypopharyngeal and laryngeal cancers, the SLFN11-positive groups exhibited a significantly superior PFS compared to the SLFN11-negative groups (5-year PFS: 71.2% vs. 34.9%, respectively, *p* = 0.002, **Figure 2B**; 5-year PFS: 91.7% vs. 62.5%, respectively, *p* = 0.023, **Figure 2C**; 5-year PFS: 76.7% vs. 57.7%, respectively, *p* = 0.014, **Figure 2D**). We further analyzed PFS in the intra-arterial and intravenous cisplatin groups. In both treatment groups, the SLFN11-positive groups exhibited a significantly superior PFS than the SLFN11-negative groups (5-year PFS: 74.7% vs. 42.4%, respectively, *p* = 0.002, **Figure 2E**; 5-year PFS: 82.9% vs. 61.3%, respectively, *p* = 0.002, **Figure 2F**). Univariate analysis revealed that the primary site (*p* = 0.008), T classification (*p* = 0.017), p53 status (*p* = 0.049), and SLFN11 status (*p* < 0.001) were significant factors for PFS in all cases (**Table 2**). Moreover, in multivariate analysis, the SLFN11 status was the only independent factor affecting PFS (*p* < 0.001). Additionally, the interaction term “primary site*SLFN11 status” was not significant (**Supplementary Table 2**).

**Table 2:** Univariate and multivariate Cox proportional hazards analysis of progression-free survival in HNSCC.

Variables	Number	Univariate analysis		Multivariate analysis	
		HR (95% CI)	*p* value	HR (95% CI)	*p* value
Age (years)					
<63	81	1			
≥63	80	0.871 (0.521–1.458)	0.600		
Sex					
Male	134	1			
Female	27	1.037 (0.524–2.049)	0.917		
KPS					
90, 100	145	1			
70, 80	16	1.369 (0.620–3.018)	0.436		
Primary site					
Nasal cavity and paranasal sinus	43	1		1	
Oropharynx (p16-positive)	40	0.332 (0.147–0.750)	0.008	0.464 (0.163–1.320)	0.150
Oropharynx (p16-negative), Hypopharynx, larynx	78	0.690 (0.395–1.207)	0.193	0.696 (0.313–1.547)	0.374
Clinical stage					
II	24	1			
III, IV	137	1.631 (0.700–3.796)	0.256		
T classification					
T1–2	63	1		1	
T3–4	98	2.004 (1.128–3.561)	0.017	1.795 (0.881–3.657)	0.107
N classification					
N0	78	1			
N1–3	83	1.060 (0.635–1.770)	0.822		
Histological grade					
Well-Moderate	108	1			
Poor	53	0.740 (0.417–1.316)	0.306		
Cisplatin injection					
Intra-arterial	69	1		1	
Intravenous	92	0.631 (0.378–1.054)	0.078	1.152 (0.512–2.593)	0.731
Ki-67					
Negative	65	1			
Positive	96	0.964 (0.573–1.623)	0.892		
p53					
NE	57	1		1	
EP, EN	104	1.800 (1.001–3.236)	0.049	1.525 (0.816–2.849)	0.185
SLFN11					
Negative	80	1		1	
Positive	81	0.311 (0.176–0.548)	<0.001	0.288 (0.161–0.514)	<0.001

HR, hazard ratio; CI, confidence interval.

### 3.3 The SLFN11-positive group exhibited better OS than the SLFN11-negative group

OS curves were calculated using the Kaplan–Meier method and compared by the log-rank test. The OS of the patients in the SLFN11-positive group was significantly better than that in the SLFN11-negative group (5-year OS: 89.9% vs. 80.2%, respectively, *p* = 0.002, **Supplementary Figure 1A**). Next, we performed an in-depth analysis of the primary sites. In nasal cavity and paranasal sinus cancers, the SLFN11-positive groups exhibited a significantly superior OS compared to the SLFN11-negative groups (5-year PFS: 86.4% vs. 74.8%, respectively, *p* = 0.031, **Supplementary Figure 1B**). However, there were no significant differences in other primary regions (**Supplementary Figure 1C, 1D**).

We further analyzed OS in the intra-arterial and intravenous cisplatin groups. In both treatment groups, the SLFN11-positive groups exhibited a significantly superior OS than the SLFN11-negative groups (5-year OS: 84.6% vs. 76.4%, respectively, *p* = 0.038, **Supplementary Figure 1E**; 5-year PFS: 93.7% vs. 83.1%, respectively, *p* = 0.039, **Supplementary Figure 1F**). Multivariate analysis revealed that SLFN11 status was the only independent factor affecting OS in all cases (*p* = 0.005, **Supplementary Table 3**). Additionally, the interaction term “primary site*SLFN11 status” was not significant (**Supplementary Table 4**).

### 3.4 SLFN11 expression ratio is decreased in residual or recurrent tumors after CRT

**Figure 3:**
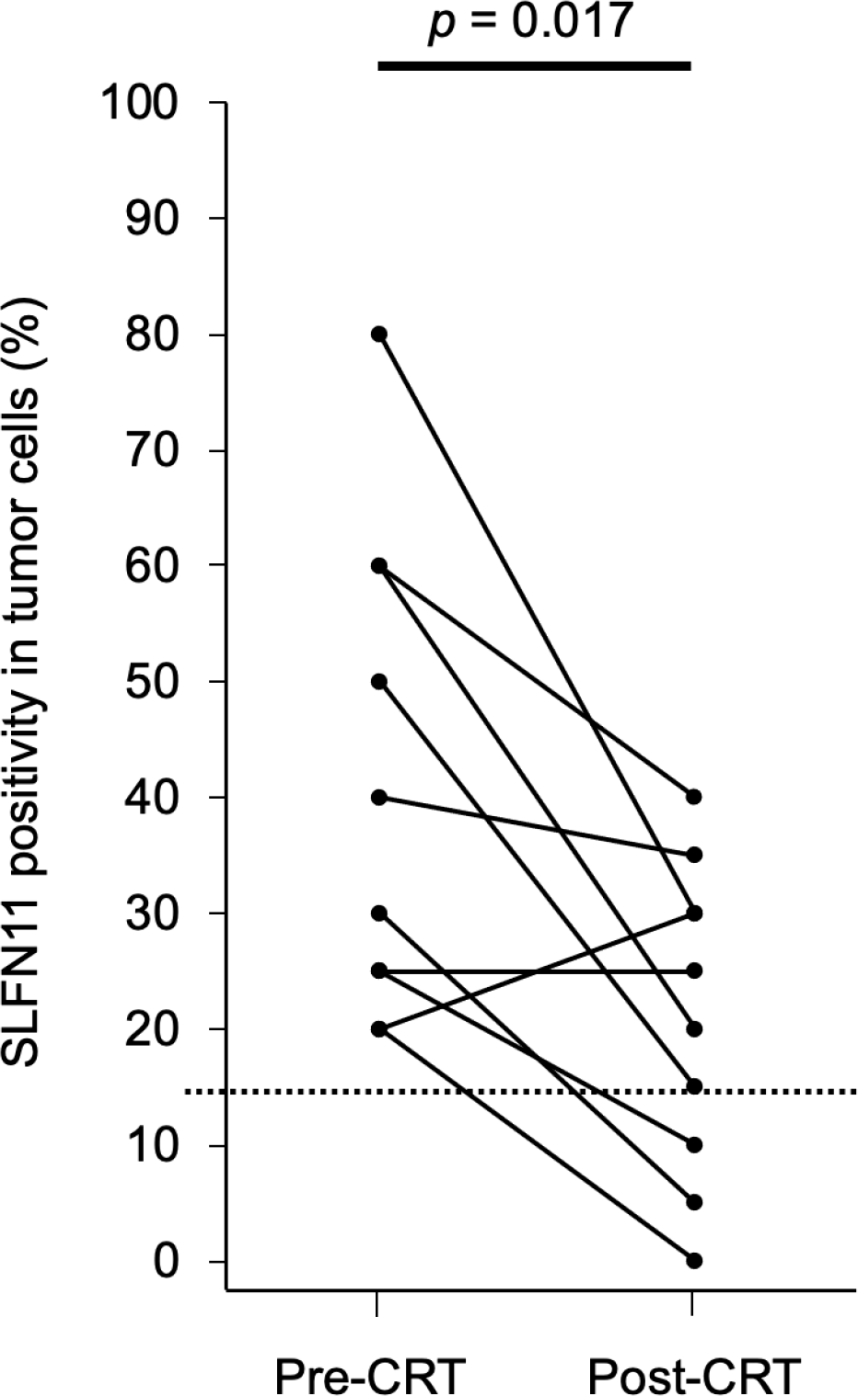
Percentage of SLFN11 positivity in tumor cells before and after CRT. SLFN11 expression in tumors before CRT and in residual or recurrent tumors with local failure after CRT is plotted for 10 paired samples. The dashed line represents the threshold value (15%) used for defining SLFN11-positive samples. Data were analyzed using the Wilcoxon signed-rank test.

To examine changes in SLFN11 expression in residual or recurrent tumors, we selected 10 residual or recurrent cases that were SLFN11-positive before CRT. We then compared SLFN11 expression in these paired samples before and after CRT. The percentage of SLFN11 positivity was significantly decreased in residual or recurrent tumors after CRT in 8 of 10 cases (*p* = 0.017, **Figure 3**). In contrast, there was no significant difference in the percentage of Ki-67 positivity before and after CRT (*p* = 0.324).

### 3.5 SLFN11 expression determines cisplatin sensitivity in HNSCC cell lines

To assess the impact of SLFN11 on the sensitivity to platinum-based CRT in HNSCC, we performed *in vitro* experiments using HNSCC cell lines. First, we checked *SLFN11* mRNA expression by quantitative RT-PCR in five HNSCC cell lines (HSC-2, HSC-3, HSC-4, SCC-9, and SCC-25) and found that *SLFN11* expression in HSC-2, HSC-3, and HSC-4 cells was higher than that in SCC-9 and SCC-25 cells (**Supplementary Figure 2A**). Comparable results were obtained in immunoblotting analysis (**Supplementary Figure 2B**). DU145 parental and *SLFN11*-KO cells, which we generated previously (4), were used as positive and negative controls, respectively. Next, we evaluated the sensitivity to cisplatin in HNSCC cell lines by performing a cell viability assay (**Supplementary Figure 2C**). The IC_50_ values of cisplatin after 72 h were 4.42, 0.69, 1.52, 22.91, and 11.44 μM in HSC-2, HSC-3, HSC-4, SCC-9, and SCC-25 cells, respectively. Overall, the IC_50_ values in HNSCC cell lines with high *SLFN11* expression were lower than those in cell lines with low *SLFN11* expression. Similar results were obtained when carboplatin (a platinum analog) was used. In contrast, the sensitivity to the non-DNA-damaging drug docetaxel, which is widely used for induction chemotherapy in HNSCC (21), was not altered, regardless of the expression of *SLFN11* in the five cell lines.

### 3.6 SLFN11-KO reduces sensitivity to cisplatin in HNSCC cell lines

**Figure 4:**
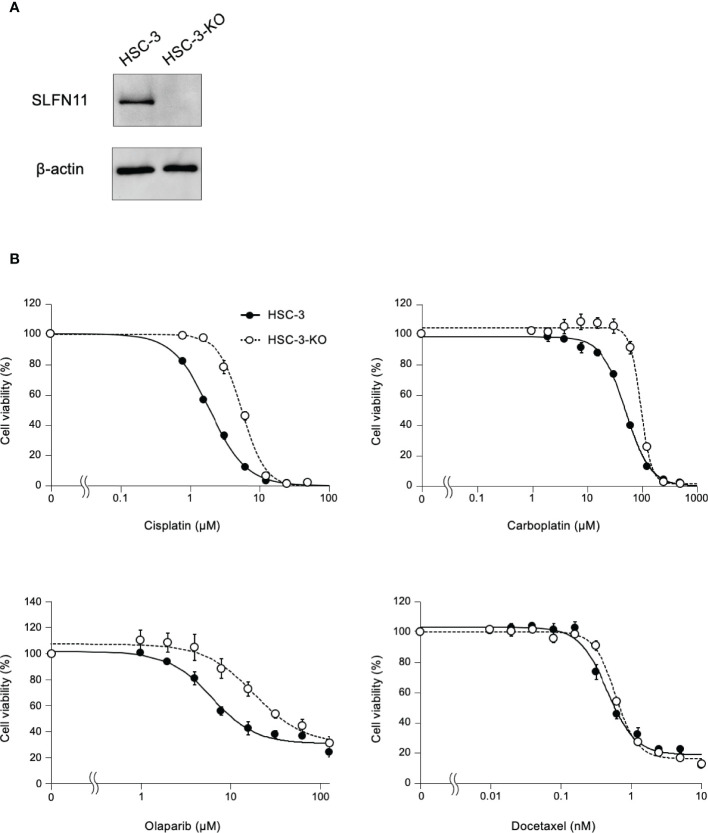
HSC-3 cells deficient in SLFN11 display reduced sensitivity to cisplatin, carboplatin, and olaparib. **(A)** *SLFN11*-KO cells from HSC-3 cells (HSC-3-KO) were generated using the CRISPR/Cas9 method. Cell lysates prepared from these cells were subjected to immunoblot analysis with an antibody for SLFN11 and β-actin (loading control). **(B)** HSC-3-KO and parental HSC-3 cells were exposed to cisplatin and carboplatin for 48 h, or olaparib and docetaxel for 72 h at the indicated concentrations. Cell viability was determined using a Cell Counting Kit-8 assay and the values were normalized to those of untreated cells. Data are presented as the mean ± standard error of the mean from three independent experiments.

To further determine whether the sensitivity to cisplatin depends on SLFN11 expression, we established *SLFN11*-KO isogenic cell lines from HSC-3 and HSC-4 cells using CRISPR/Cas9, which were named as HSC-3-KO and HSC-4-KO, respectively (**Figure 4A**; **Supplementary Figure 3A**). In the absence of cisplatin, the growth rate did not differ between the KO and corresponding parental cells for both strains (**Supplementary Figure 4**). The cell viability assay showed that HSC-3-KO and HSC-4-KO cells exhibited reduced sensitivity to cisplatin compared to their parental counterparts (**Figure 4B**; **Supplementary Figure 3B**). The IC_50_ values of cisplatin after 48 h between HSC-3-KO vs. HSC-3 and HSC-4-KO vs. HSC-4 were 5.56 vs. 1.91 μM and 10.75 vs. 4.18, respectively. Similar results were obtained using carboplatin and olaparib, a clinically approved poly (ADP-ribose) polymerase inhibitor. HSC-3-KO and HSC-4-KO cells did not show increased resistance to docetaxel compared to their parental counterparts.

## 4 Discussion

We identified SLFN11 as a novel biomarker for predicting the therapeutic outcomes of platinum-based CRT in HNSCC. To the best of our knowledge, this is the first study to analyze the role of SLFN11 in the response to CRT using clinical HNSCC samples as well as multiple and isogenic HNSCC cell lines.

In p16-positive OPC, patients with SLFN11-positive tumors showed a better PFS than those with SLFN11-negative tumors; however, no significant differences were observed in OS. Consistent results were also obtained in the p16-negative oropharyngeal, hypopharyngeal, and laryngeal cancers. The lack of significant difference in OS regardless of the differences in PFS in these regions was attributed to the salvage surgery in patients with these cancers, which tended to achieve good results despite disease progression following CRT (22, 23). As for p16-positive OPC, adjuvant chemotherapy, salvage surgery, and CRT have been associated with a good prognosis (24, 25).

Interestingly, the percentage of SLFN11 positivity was significantly higher in patients with p16-positive OPC than in those with p16-negative OPC. A similar result was observed for The Cancer Genome Atlas HNSCC cohort by Lee et al. (26). By analyzing RNA sequencing data, they found little correlation between the *SLFN11* expression and *SLFN11* copy number. However, the relationship between human papillomavirus (HPV) infection and SLFN11 expression remains unclear. Given that SLFN11 is known to be induced by interferon stimulation and thereby plays a role in antiviral responses (6), SLFN11 activation by HPV infection may increase the sensitivity to CRT in p16-positive OPC.

We further demonstrated that SLFN11 expression was decreased in residual or recurrent tumors after CRT. Gardner et al. reported that patient-derived xenografts of small cell lung cancer acquired chemoresistance to DNA-damaging agents (cisplatin and etoposide regimens) through epigenetic silencing of *SLFN11* (27). Additionally, Takashima et al. reported that a gastric cancer cell line acquired resistance to oxaliplatin (a platinum derivative) by loss of SLFN11 expression following continuous oxaliplatin treatment (9). The findings of our study may be explained by a similar mechanism. However, given that the intensity of SLFN11 expression was highly heterogeneous in some tumor tissues before CRT (**Supplementary Figure 5**), it is possible that a population of cells with low SLFN11 expression in the primary tumors may have survived and proliferated after CRT.

We confirmed the relationship between SLFN11 expression and cisplatin sensitivity at the cellular level using HNSCC cell lines. In addition, SLFN11 expression was found to be associated with the response to another platinum-based drug (carboplatin) and poly (ADP-ribose) polymerase inhibitor (olaparib), although it was not associated with the response to a non-DNA-damaging agent (docetaxel). Similar results were obtained in other cancer cell lines. For example, Zoppoli et al. reported that following treatment with taxol and staurosporine, there was no significant difference in cell viability between *SLFN11-*knockdown and control prostate cancer cells (3). Thus, the function of SLFN11 in sensitizing HNSCC cells to anticancer drugs is specific to DNA-damaging agents.

We previously reported that SLFN11 is recruited to stressed replication forks through replication protein A, and induced replication arrest by blocking the replicative helicase complex (28). Subsequent studies reported the role of SLFN11-dependent tRNA-cleavage in insufficient ATR synthesis, chromatin opening, degradation of the replication initiation factor CDT1, and degradation of reversed replication forks (7, 8, 28, 29). However, these studies were mostly conducted using camptothecin (topoisomerase I inhibitor) and not cisplatin, but given that replication stress is a stimulatory signal for SLFN11 expression, the same results can be expected with cisplatin. Moreover, the structure of full-length SLFN11 was recently elucidated by cryo-electron microscopy (30). Further, the structural interaction between SLFN11 and single-strand DNA and tRNA cleavage by SLFN11 was clarified; therefore, the mechanism underlying the function of SLFN11 can be further elucidated.

The sensitivity to cisplatin can be predicted by examining the expression of SLFN11 *via* immunohistochemical analysis prior to CRT; this prediction can facilitate the selection of more effective treatments for patients with HNSCC. However, there is no consensus on the appropriate threshold for SLFN11 expression. Pietanza et al. used the H-score, a product of the percentage of positive tumor cells and staining intensity, and defined a cutoff of ≥1 for SLFN11-positive cases in small cell lung cancer (11).Takashima et al. classified the SLFN11 immunohistochemical staining profiles into three groups, 1+ (1%–10%), 2+ (11%–50%), and 3+ (51%–100%), for semiquantitative evaluation of SLFN11 expression across a wide variety of sections of normal and tumor tissues (16). For clinical application, further studies are required to determine the optimal positive threshold for SLFN11 expression in HNSCC.

This study has some limitations. First, we reviewed patients treated with CRT, not with cisplatin monotherapy. Radiation causes DNA damage; hence, SLFN11 expression may correlate with the sensitivity of cancer cells to radiation therapy as well as cisplatin. We are planning a clinical study in which HNSCC patients treated with radiotherapy alone and cell viability assays treated with radiation. Second, this clinical study is retrospective and the threshold for SLFN11 expression was set following data collection. To overcome this limitation, further studies with a prospective design are required.

In conclusion, our clinical and experimental findings demonstrate a correlation between SLFN11 expression and cisplatin sensitivity in HNSCC. SLFN11 is useful for predicting cisplatin sensitivity in HNSCC and can contribute to the development of precision medicine.

## Data availability statement

The raw data supporting the conclusions of this article will be made available by the authors, without undue reservation.

## Ethics statement

The studies involving human participants were reviewed and approved by Institutional Review Board of Hokkaido University Hospital (No. 020-0055). The patients/participants provided their written informed consent to participate in this study.

## Author contributions

The authors confirm contribution to the paper as follows: conceptualization: SH, SK, JM, and MS. data curation: SH and TM. formal analysis: SH and TS. funding acquisition: SK, TM, MS, YO, and AH. investigation: SH, JM, TS, TT, DT, NS, and YF. methodology: SH, SK, and MS; project administration: SH, SK, YO, and AH. resources: SH, SK, JM, NT, YF, YO, and AH. software: SH and TS. supervision: SK, YO, and AH. validation: SH, SK, JM, TT, DT, and NS. visualization: SH. writing - original draft: SH and SK. writing - review and editing: SH, SK, JM, NT, MS, YF, YO, and AH. All authors contributed to the article and approved the submitted version.
