# Supplementary material for: Schlafen family member 11 indicates favorable prognosis of patients with head and neck cancer following platinum-based chemoradiotherapy
Source: Front Oncol. 2023 Jan 19;12:978875. doi: 10.3389/fonc.2022.978875 (PMC9892834; doi:10.3389/fonc.2022.978875)
Supplement: Supplementary file 1 [file DataSheet_1.docx]

Supplementary Material

**Supplementary Tables**

**Supplementary Table 1. Relation between SLFN11 and p16 status in OPC**

|  | No. of patients | | | | |  |
| --- | --- | --- | --- | --- | --- | --- |
|  | | All | SLFN11-positive | SLFN11-negative | *p* value | |
| p16-positive | | 40 | 24 | 16 | 0.030 | |
| p16-negative | | 21 | 6 | 15 |  | |

**Supplementary Table 2. Multivariate Cox proportional hazards analysis including an interaction term (primary site*SLFN11 status) of progression-free survival**

| Variables |  | HR (95% CI) | *p* value |  |
| --- | --- | --- | --- | --- |
| Primary site |  |  |  |  |
| Nasal cavity and paranasal sinus |  | 1 |  |  |
| Oropharynx (p16-positive) |  | 0.475 (0.151–1.488) | 0.201 |  |
| Oropharynx (p16-negative), Hypopharynx, larynx |  | 0.611 (0.254–1.470) | 0.271 |  |
| T classification |  |  |  |  |
| T1–2 |  | 1 |  |  |
| T3–4 |  | 1.764 (0.865–3.595) | 0.118 |  |
| Cisplatin injection |  |  |  |  |
| Intra-arterial |  | 1 |  |  |
| Intravenous |  | 1.151 (0.513–2.584) | 0.732 |  |
| p53 |  |  |  |  |
| NE |  | 1 |  |  |
| EP, EN |  | 1.544 (0.827–2.882) | 0.172 |  |
| SLFN11 |  |  |  |  |
| Negative |  | 1 |  |  |
| Positive |  | 0.240 (0.095–0.608) | 0.002 |  |
| Primary site*SLFN11 interaction |  |  |  |  |
| Nasal cavity and paranasal sinus |  | 1 |  |  |
| Oropharynx (p16-positive) |  | 0.869 (0.136–5.557) | 0.882 |  |
| Oropharynx (p16-negative), Hypopharynx, larynx |  | 1.532 (0.448–5.233) | 0.496 |  |

**Supplementary Table 3. Univariate and multivariate Cox proportional hazards analysis of overall survival in HNSCC**

|  | Number |  | Univariate analysis | |  | Multivariate analysis | |
| --- | --- | --- | --- | --- | --- | --- | --- |
| Variables |  |  | HR (95% CI) | *p* value |  | HR (95% CI) | *p* value |
| Age (years) |  |  |  |  |  |  |  |
| <63 | 81 |  | 1 |  |  |  |  |
| ≥63 | 80 |  | 1.225 (0.618–2.428) | 0.560 |  |  |  |
| Sex |  |  |  |  |  |  |  |
| Male | 134 |  | 1 |  |  |  |  |
| Female | 27 |  | 1.072 (0.443–2.589) | 0.877 |  |  |  |
| KPS |  |  |  |  |  |  |  |
| 90, 100 | 145 |  | 1 |  |  |  |  |
| 70, 80 | 16 |  | 1.048 (0.319–3.442) | 0.938 |  |  |  |
| Primary site |  |  |  |  |  |  |  |
| Nasal cavity and paranasal sinus | 43 |  | 1 |  |  | 1 |  |
| Oropharynx (p16-positive) | 40 |  | 0.290 (0.080–1.042) | 0.057 |  | 0.377 (0.101–1.412) | 0.147 |
| Oropharynx (p16-negative), Hypopharynx, larynx | 78 |  | 0.983 (0.470–2.053) | 0.964 |  | 1.129 (0.501–2.546) | 0.769 |
| Clinical stage |  |  |  |  |  |  |  |
| II | 24 |  | 1 |  |  |  |  |
| III, IV | 137 |  | 1.231 (0.433–3.498) | 0.696 |  |  |  |
| T classification |  |  |  |  |  |  |  |
| T1–2 | 63 |  | 1 |  |  | 1 |  |
| T3–4 | 98 |  | 1.941 (0.905–4.160) | 0.088 |  | 1.864 (0.805–4.311) | 0.145 |
| N classification |  |  |  |  |  |  |  |
| N0 | 78 |  | 1 |  |  |  |  |
| N1–3 | 83 |  | 1.272 (0.642–2.520) | 0.490 |  |  |  |
| Histological grade |  |  |  |  |  |  |  |
| Well-Moderate | 108 |  | 1 |  |  |  |  |
| Poor | 53 |  | 0.730 (0.340–1.568) | 0.420 |  |  |  |
| Cisplatin injection |  |  |  |  |  |  |  |
| Intra-arterial | 69 |  | 1 |  |  |  |  |
| Intravenous | 92 |  | 0.719 (0.366–1.415) | 0.340 |  |  |  |
| Ki-67 |  |  |  |  |  |  |  |
| Negative | 65 |  | 1 |  |  |  |  |
| Positive | 96 |  | 0.772 (0.393–1.516) | 0.453 |  |  |  |
| p53 |  |  |  |  |  |  |  |
| NE | 57 |  | 1 |  |  |  |  |
| EP, EN | 104 |  | 1.325 (0.632–2.772) | 0.455 |  |  |  |
| SLFN11 |  |  |  |  |  |  |  |
| Negative | 80 |  | 1 |  |  | 1 |  |
| Positive | 81 |  | 0.341 (0.162–0.714) | 0.004 |  | 0.349 (0.165–0.737) | 0.005 |
|  |  |  |  |  |  |  |  |

**Supplementary Table 4. Multivariate Cox proportional hazards analysis including an interaction term (primary site*SLFN11 status) of overall survival**

| Variables |  | HR (95% CI) | *p* value |  |
| --- | --- | --- | --- | --- |
| Primary site |  |  |  |  |
| Nasal cavity and paranasal sinus |  | 1 |  |  |
| Oropharynx (p16-positive) |  | 0.424 (0.156–1.153) | 0.092 |  |
| Oropharynx (p16-negative), Hypopharynx, larynx |  | 0.636 (0.305–1.324) | 0.226 |  |
| T classification |  |  |  |  |
| T1–2 |  | 1 |  |  |
| T3–4 |  | 1.628 (0.853–3.108) | 0.139 |  |
| SLFN11 |  |  |  |  |
| Negative |  | 1 |  |  |
| Positive |  | 0.256 (0.102–0.647) | 0.003 |  |
| Primary site*SLFN11 interaction |  |  |  |  |
| Nasal cavity and paranasal sinus |  | 1 |  |  |
| Oropharynx (p16-positive) |  | 0.836 (0.131–5.345) | 0.850 |  |
| Oropharynx (p16-negative), Hypopharynx, larynx |  | 1.452 (0.426–4.940) | 0.551 |  |

**Supplementary Figures**


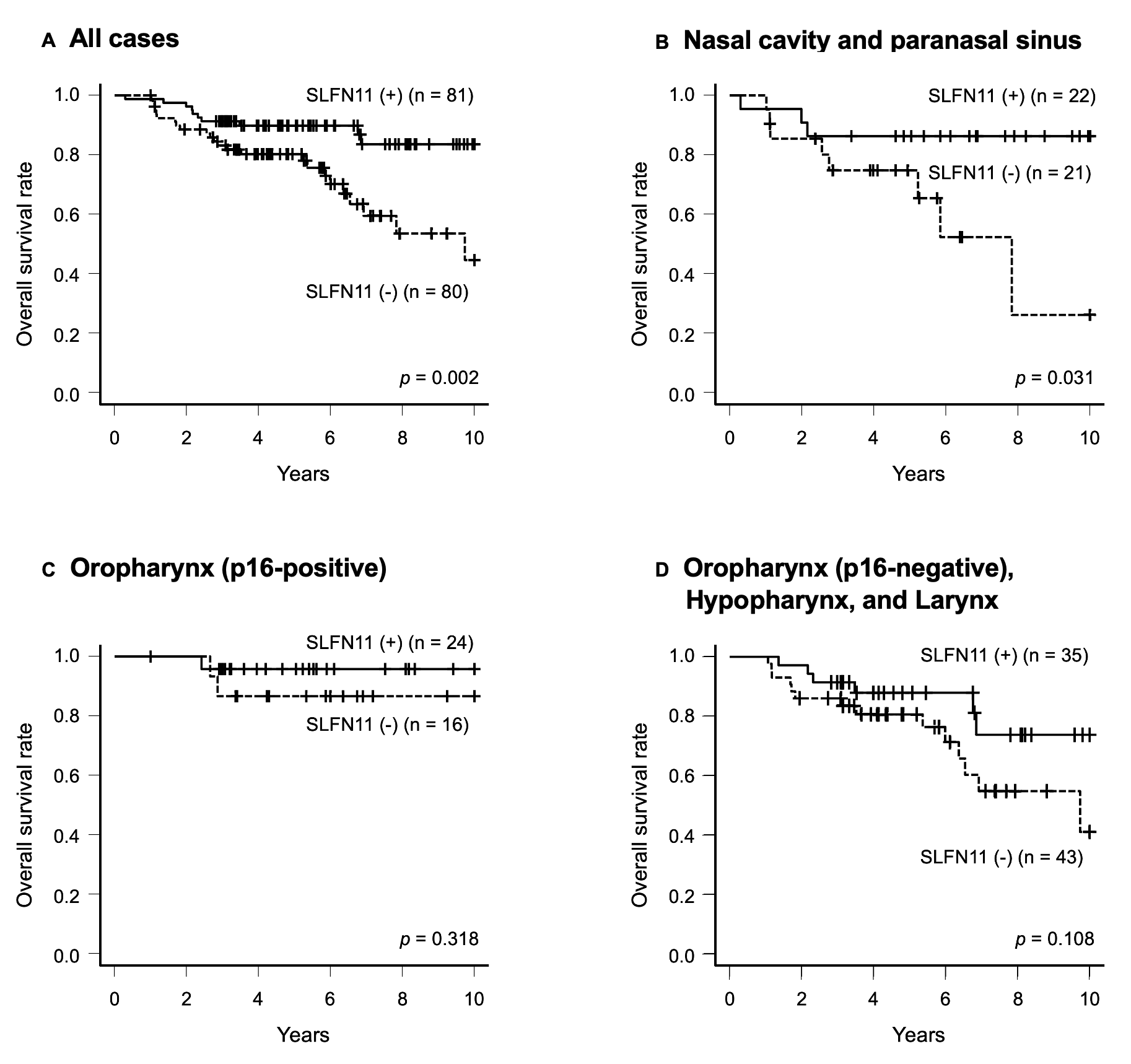


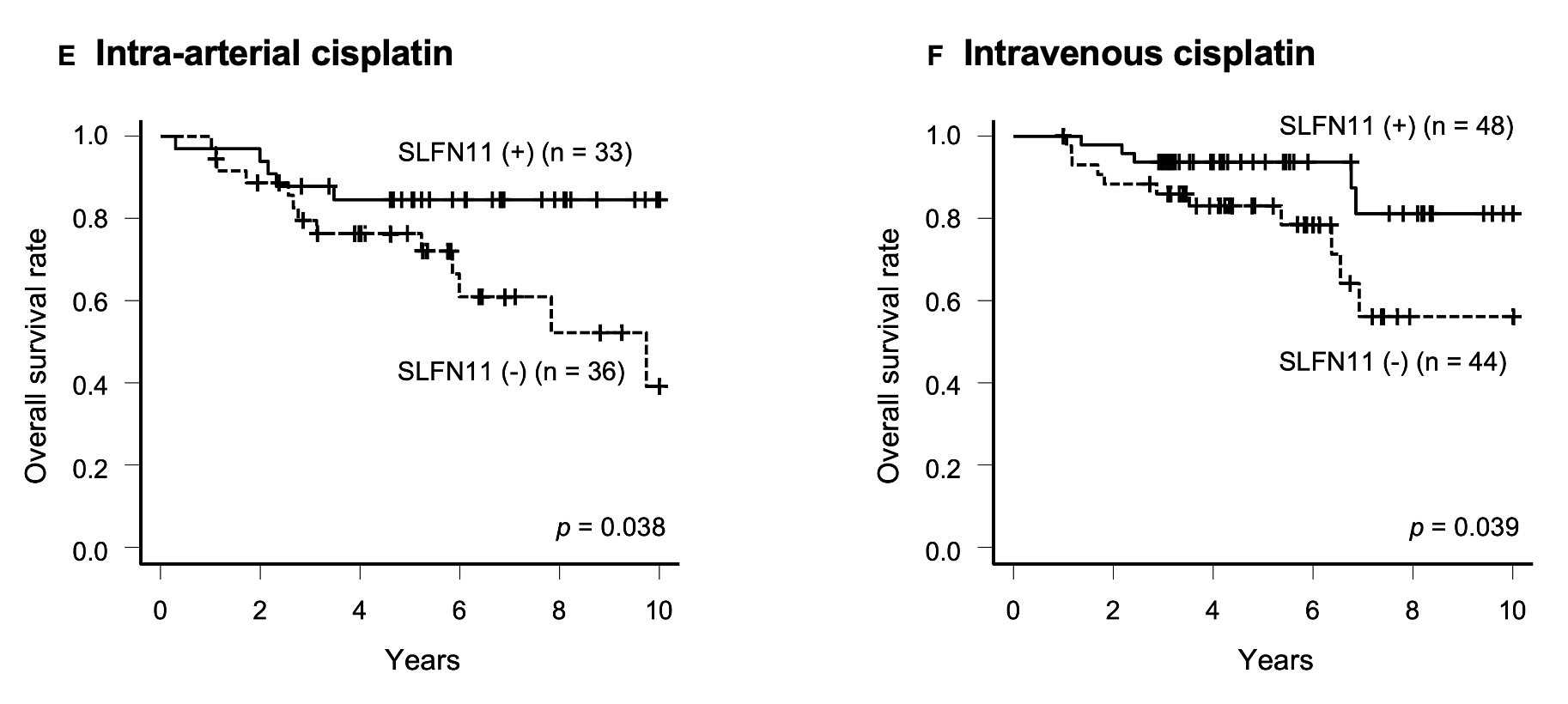


**Supplementary Figure 1. Overall survival (OS) in patients with SLFN11-positive HNSCC**

OS curves generated by the Kaplan–Meier method are shown for all 161 patients in **(A)** and those with tumors arising from each primary site are indicated at the top of the panel in **(B–D)**. Intra-arterial and intravenous cisplatin were analyzed separately in **(E, F)**. The difference between groups was calculated using the log-rank test.

**
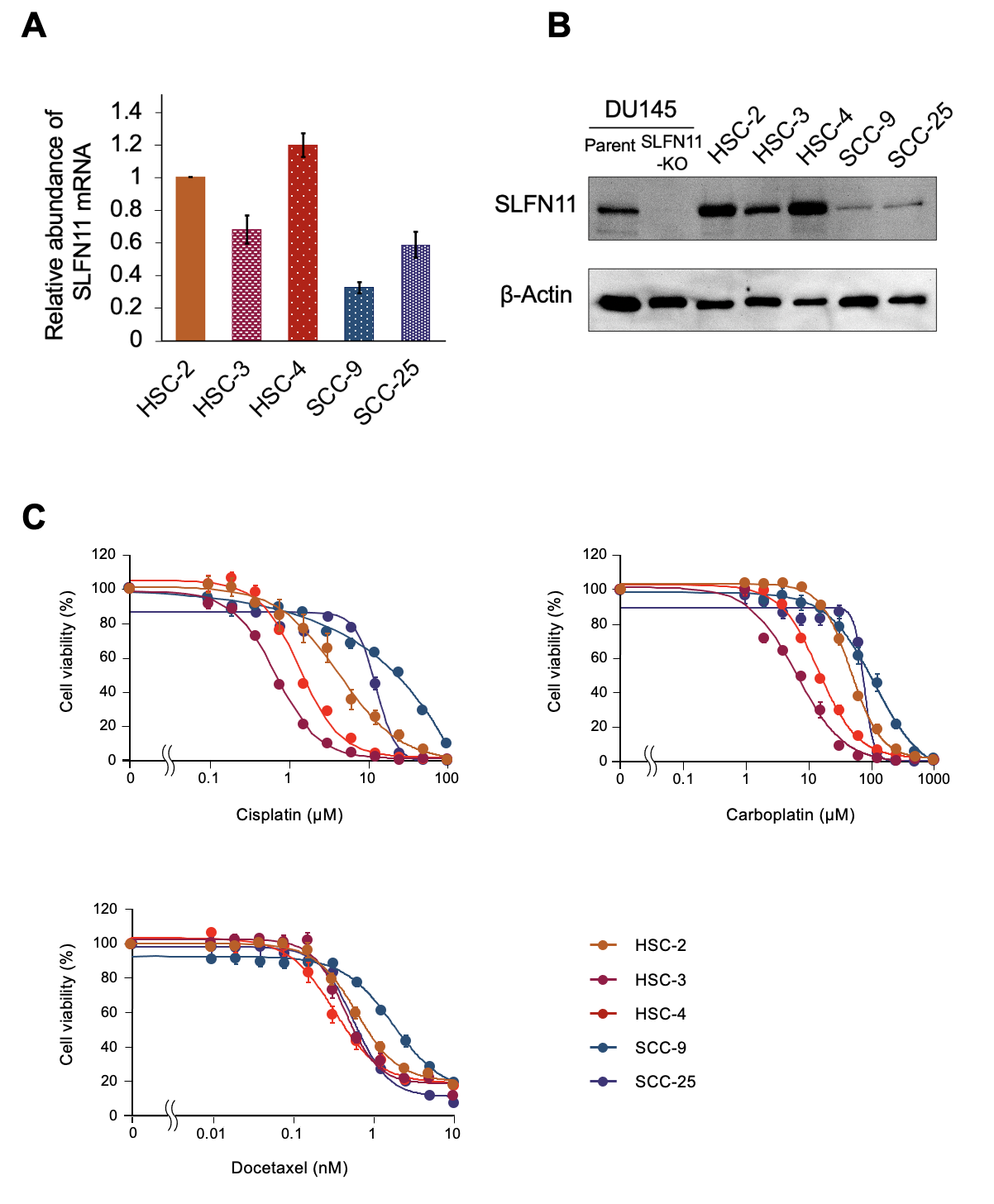
**

**Supplementary Figure 2. SLFN11 expression and drug sensitivity in HNSCC cell lines**

**(A)** mRNA levels of *SLFN11* in HNSCC cell lines were analyzed using quantitative RT-PCR and normalized to that of *GAPDH*. The values obtained for each cell line were further normalized to those for control HSC-2 cells. Data are presented as the mean ± standard error of the mean (SEM) from three independent experiments. **(B)** Protein levels of SLFN11 were analyzed using immunoblotting. DU145 parental and *SLFN11*-KO cells were used as the positive and negative control, respectively. **(C)** Cells indicated on the right were exposed to the indicated doses of cisplatin, carboplatin, and docetaxel for 72 h, and the number of cells was determined using the Cell Counting Kit-8 assay. The values obtained were normalized to those for untreated cells and plotted. Data are presented as the mean ± SEM from three independent experiments.


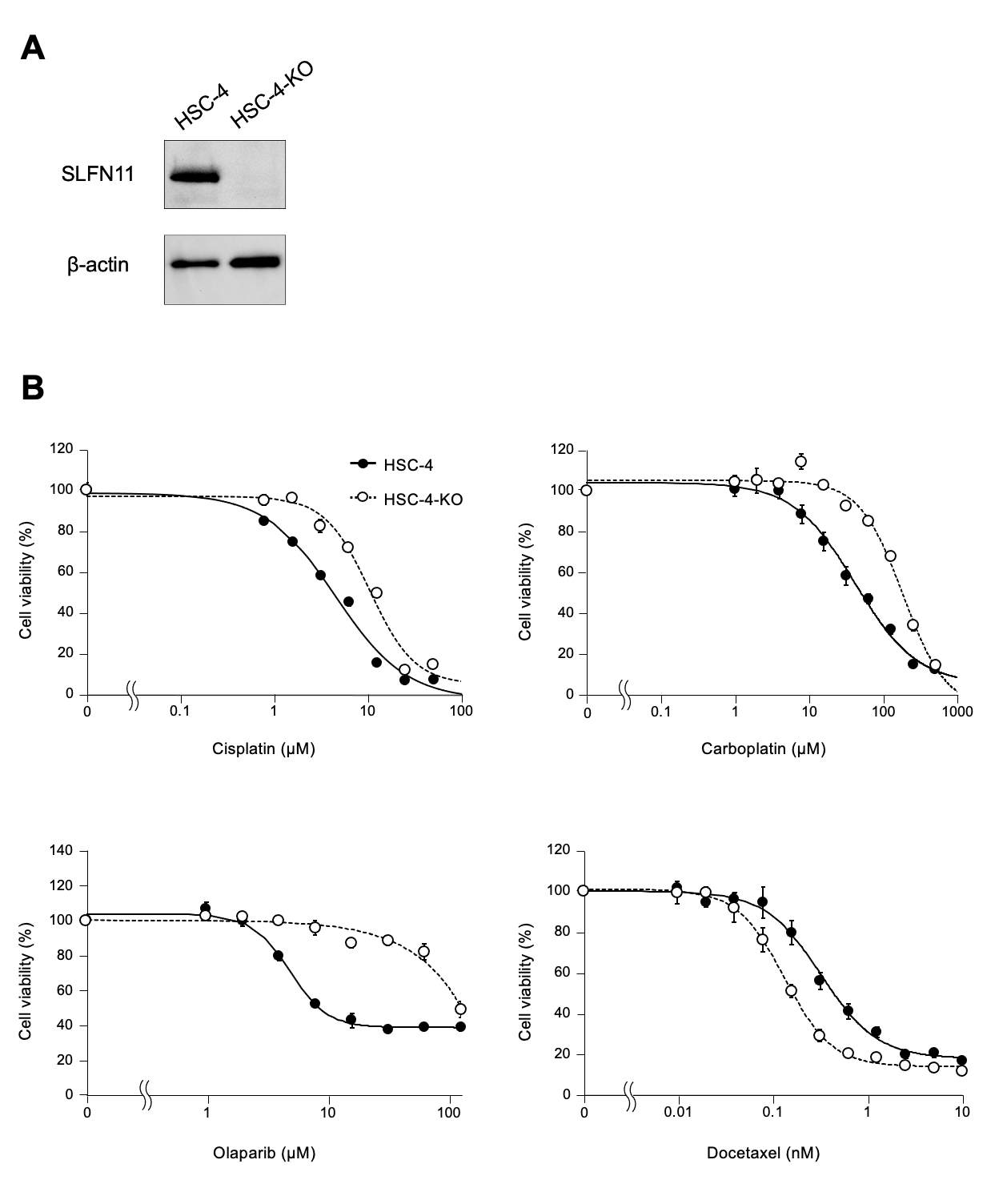


**Supplementary Figure 3. Effect of *SLFN11*-KO on drug sensitivity in HSC-4 cells**

**(A)** HSC-4 cells deficient in *SLFN11* (HSC-4-KO) were generated as described in Figure 4. SLFN11 protein level was confirmed using immunoblotting. **(B)** Cell viability after exposure to each drug at the indicated concentrations was determined as depicted in Figure 4B and plotted. Data are presented as the mean ± standard error of the mean from three independent experiments.


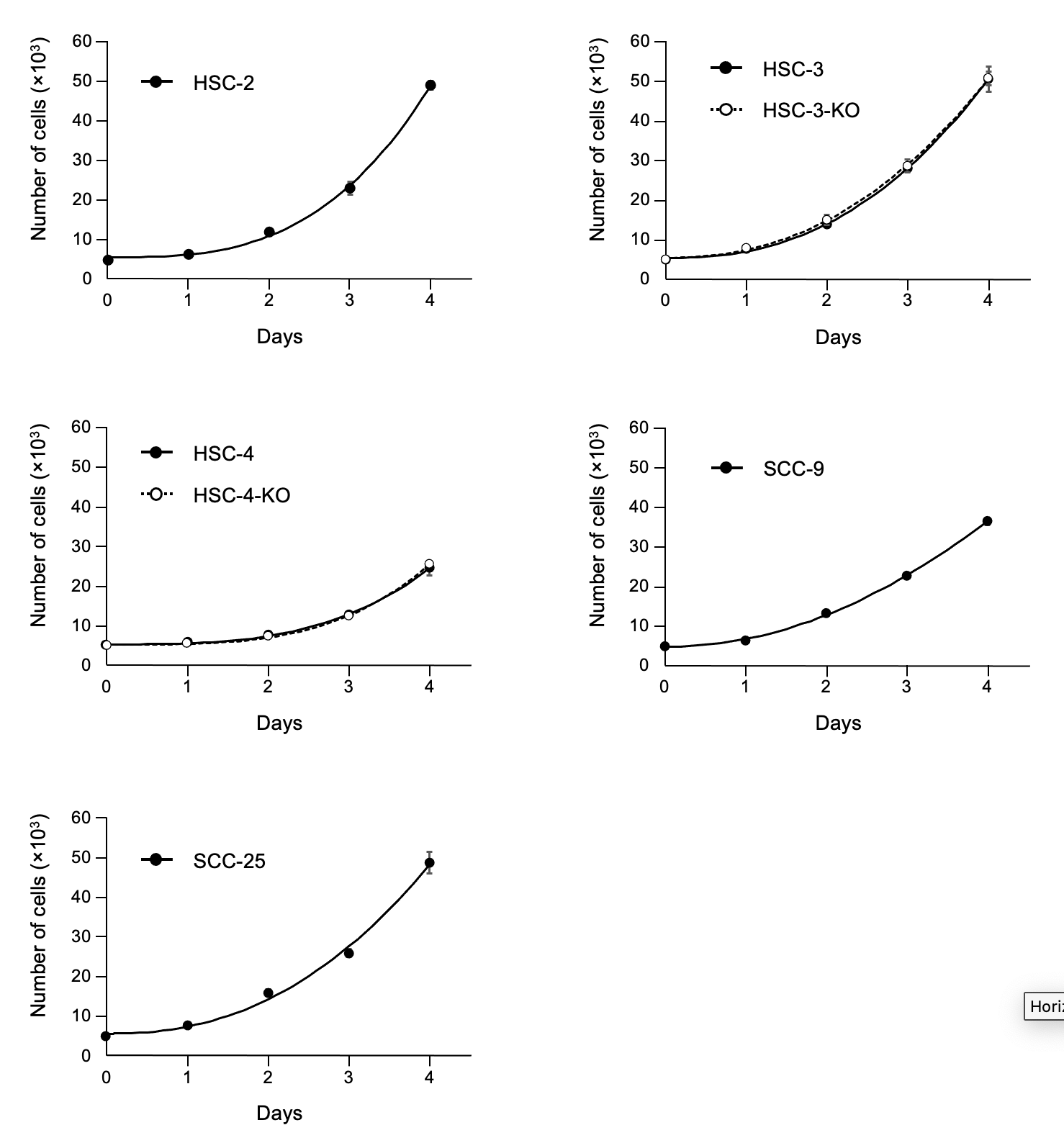


**Supplementary Figure 4. Effect of *SLFN11*-KO on cell proliferation in HNSCC cell lines**

The number of cells was counted every day for up to 4 days and plotted. Data are presented as the mean ± standard error of the mean from three independent experiments.


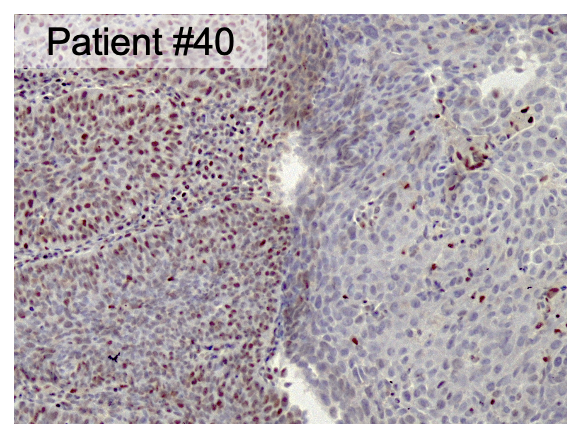


**Supplementary Figure 5. Intratumor heterogeneity in SLFN11 expression**

The representative image in which SLFN11 expression displayed a heterogeneous pattern within the tumor are presented. (Patient #40): an immunohistochemical microgram of T2N2b hypopharyngeal cancer treated with CRT with intravenous cisplatin. In this case, nearly all cancer cells on the left side of the photo are positive for SLFN11, whereas on the right side, there are almost no SLFN11-positive cells. The overall positive ratio of SLFN11 was 55%.
